# Multi-dimensional epidemiology of pediatric acute respiratory tract infection in Ningbo, China (2020–2024): age-specific susceptibility, pathogen dynamics, and epidemiological trends

**DOI:** 10.3389/fcimb.2026.1662777

**Published:** 2026-02-02

**Authors:** Min Jiang, Qian Sun, Xuedan Qiu, Qianru Mei, Yanye Tu, Feng Wang, Qingcao Li

**Affiliations:** Department of Clinical Laboratory, The Affiliated Lihuili Hospital of Ningbo University, Ningbo, China

**Keywords:** acute respiratory tract infection, epidemic characteristics, Ningbo, pathogen, pediatric

## Abstract

**Background:**

Pediatric acute respiratory tract infection (ARTI) constitutes a major global health threat. Since 2020, global containment measures have disrupted pathogen circulation patterns, leading to altered epidemiological profiles across multiple pathogens with marked regional heterogeneity. Current research in Ningbo predominantly focuses on single-pathogen surveillance or short-term monitoring, lacking systematic analysis of multi-pathogen interactive dynamics, age-specific susceptibility mechanisms, and co-infections.

**Methods:**

Clinical data from all 191,967 pediatric patients with ARTI at Ningbo LiHuili Hospital (2020–2024) were collected. Laboratory testing including influenza A/B virus (IFV-A/B) detection, *Mycoplasma pneumoniae* (MP) testing, 13 respiratory pathogens analysis, and bacterial culture was systematically integrated and analyzed.

**Results:**

From 2020 and 2024, the number of ARTI cases initially increased and then decreased, peaking in 2023. The incidence was highest in autumn and lowest in summer. Among all cases, 75,001 (39.07%) were diagnosed with acute lower respiratory infection (ALRI), with children aged 6 years exhibiting the highest susceptibility. Of the 41,766 cases tested, the overall pathogen detection rate was 67.61%. IFV-A/B, Human Parainfluenza Virus (HPIV), and Human Adenovirus (HAdV) were more frequently detected in acute upper respiratory tract infections (AURI) (*P* < 0.05), while Human Rhinovirus (HRV), Respiratory Syncytial Virus (RSV), and MP were predominantly identified in ALRI cases (*P* < 0.05). Each pathogen exhibited age-specific susceptibility patterns. Several pathogens, such as IFV-A, MP, and HAdV, displayed distinctive epidemic peaks. Co-infections were common, with a 17.38% co-infection rate observed in the group tested for 13 respiratory pathogens, and a higher co-infection rate of 39.49% when testing combined respiratory pathogens and bacteria. Correlation analysis among pathogens revealed predominant antagonistic interactions between viruses, positive associations among bacteria, and generally positive interactions between viruses and bacteria, though overall correlation strengths were weak.

**Conclusion:**

Pediatric ARTI exhibit age-specific susceptibility and pathogen variation. The 2023–2024 resurgence demands precision control strategies for optimized intervention.

## 1 Introduction

Acute respiratory tract infection (ARTI) remains one of the leading causes of morbidity and mortality among children worldwide (Li et al., 2021). It is estimated that over 40 million pediatric infections occur annually, resulting in approximately 650,000 deaths (Collaro et al., 2023). The etiological agents of pediatric ARTI are highly diverse, including viruses such as respiratory syncytial virus (RSV), influenza virus, and human rhinovirus (HRV), as well as bacteria and mycoplasma (Chen et al., 2022; Wu et al., 2023; Zhao et al., 2024). However, the clinical manifestations of infections caused by different pathogens often overlap considerably, and co-infections are frequent, contributing to diagnostic challenges and the overuse of antibiotics (Zhu et al., 2025). These circumstances highlight an urgent need for precision diagnostics and treatment.

Since the beginning of the 21st century, the world has witnessed several pandemics of respiratory infectious diseases (He et al., 2023). Among them, the coronavirus disease 2019 (COVID-19) pandemic and the associated implementation of non-pharmaceutical interventions (NPIs) significantly altered the transmission dynamics of respiratory pathogens, driving the activity levels of many pathogens to historical lows (Chow et al., 2023; Sun et al., 2024). Nonetheless, prolonged social isolation and protective measures have led to insufficient immune stimulation within populations, resulting in the phenomenon known as “immunity debt” (Baker et al., 2020; Cohen et al., 2021). As NPIs have gradually been lifted, abnormal rebounds of respiratory pathogens have been reported in various countries, including Singapore (Chan et al., 2021), Israel (Amar et al., 2022), the United States, and Germany (Singer et al., 2024). However, the timing, intensity, and composition of these rebounds have varied significantly across regions, emphasizing the critical need for localized surveillance.

To date, studies conducted in Ningbo have largely focused on individual pathogens or short-term surveillance (Jiang et al., 2024). Comprehensive analyses examining multi-pathogen dynamics, age-specific susceptibility patterns, and co-infection characteristics are lacking. In this study, we retrospectively analyzed pediatric ARTI cases from Ningbo LiHuiLi Hospital between 2020 and 2024, with a particular focus on the epidemiological features and evolving trends of respiratory infections during the post-pandemic era. To provide a basis for optimizing the prevention and control strategies and diagnosis and treatment plans of respiratory tract infections in children.

## 2 Methods

### 2.1 Study population

Clinical data were collected from pediatric patients aged 0–14 years who were diagnosed with ARTI at Ningbo Lihuili Hospital, between January 1, 2020 and December 31, 2024. ARTI was defined as the presence of new-onset symptoms such as cough, sputum production, rhinorrhea, fever, sore throat, or abnormal lung auscultation findings. The data encompassed multiple variables, including sex, age, time of infection, clinical manifestations, hospitalization status, and pathogen detection results. Exclusion criteria were as follows: (1) neonates (<28 days post-birth); (2) confirmed cases of COVID-19; (3) children with recurrent chronic respiratory infections or underlying comorbidities (immunodeficiency disorders, congenital cardiopulmonary diseases, etc.); (4) re-admitted patients and cases with incomplete medical records. A total of 191,967 clinical cases were ultimately included in the analysis.

### 2.2 Grouping

The cricoid cartilage was used as an anatomical reference point to differentiate between acute upper respiratory infections (AURI) and acute lower respiratory infections (ALRI) (Zhu et al., 2025). AURI included infections occurring above the cricoid cartilage, such as pharyngitis, rhinitis, and tonsillitis, while ALRI involved infections below the cricoid cartilage, such as bronchitis, bronchiolitis, and pneumonia. Patients were further categorized into three age groups: <3 years (infant and toddler group), 3–5 years (preschool group), and 6–14 years (school-age group). This classification was based on common pediatric developmental stages and exposure risks: the <3 years group encompasses infants and toddlers who are experiencing both rapid immune maturation and first-time exposures in community settings; the 3–5 years group represents preschool children with expanding social contacts; and the 6–14 years group comprises school-aged children with more mature immune systems. Seasonal distribution was defined as follows: spring (March to May), summer (June to August), autumn (September to November), and winter (December to February).

### 2.3 Laboratory testing

Based on clinical requirements, different laboratory tests were performed on pediatric ARTI patients. These tests are mainly carried out on patients who are suspected of having a specific pathogen infection, whose condition progresses rapidly, or who need to rule out severe infections. Four types of laboratory data were collected in this study: detection of influenza A/B viruses (IFV-A/B; Sansure Biotech, Changsha, China), detection of *Mycoplasma pneumoniae* (MP; Mole Bioscience, Jiangsu, China), detection of 13 respiratory pathogens (Health Genetech, Ningbo, China), and bacterial culture. Detection of IFV-A/B, MP, and the 13 respiratory pathogens was performed using Polymerase Chain Reaction (PCR)-based nucleic acid assays. All respiratory samples were collected during the early phase of illness (≤ 3 days after symptom onset) and included nasopharyngeal swabs, throat swabs, and nasopharyngeal aspirates. Samples are collected using swabs with villi. Pediatric nurses who have received unified training perform the operation according to the standard (insert the swab into the nasopharynx and rotate it for at least 3 times, and keep it there for 5–10 seconds), and then immediately place the swab into 3 mL of viral culture medium. These samples should be sent to the laboratory for testing within 4 hours. If immediate testing is not possible, they can be stored at 4°C for 72 hours. After thoroughly mixing the samples using a vortex mixer, nucleic acid extraction and testing are carried out according to the reagent instructions. The PCR amplification procedure and performance (sensitivity, specificity) of the kit are detailed in the Supplementary materials. The 13 respiratory pathogens tested included Influenza A virus (IFV-A, types H7N9, H1N1, H3N2, H5N2), Influenza B virus (IFV-B, types Victoria and Yamagata lineages), Human Adenovirus (HAdV, types B, C, and E), Human Bocavirus (HBoV), Human Rhinovirus (HRV), Human Parainfluenza Virus (HPIV, types 1-4), Human Coronavirus (HCoV, types 229E, OC43, NL63, and HKU1), Respiratory Syncytial Virus (RSV, subgroups A and B), Human Metapneumovirus (HMPV), *Chlamydia* (CH, including *Chlamydia trachomatis* and *Chlamydia pneumoniae*), and *Mycoplasma pneumoniae* (MP). Although IFV-A/B and MP were included in both the standalone tests and the 13 respiratory pathogens, they served complementary roles in the analysis and were not redundantly counted. H1N1 and H3N2 were treated as subtypes of IFV-A and not analyzed separately. For bacterial culture, upper respiratory tract specimens (nasal swabs, throat swabs, sputum) were inoculated onto blood agar plates and incubated at 37°C with 5% CO_2_ for 18–24 hours. Pick a single colony of medium size and evenly spread it on the sample target. Cover it with 1 μL of matrix, and after it dries, identify it using a mass spectrometer (Zhongyuan Huiji Biotechnology, Chongqing, China). The main bacterial species identified included *Streptococcus pneumoniae* (SP), *Haemophilus parainfluenzae* (HP), *Haemophilus influenzae* (HI), *Staphylococcus aureus* (SA), *Moraxella catarrhalis* (MC), *Acinetobacter baumannii* (AB), *Klebsiella pneumoniae* (KP), and *Escherichia coli* (EC).

### 2.4 Statistical analysis

All statistical analyses were performed using SPSS version 29.0 (IBM Corp., Armonk, NY, USA). Continuous variables that were not normally distributed were described as medians and interquartile ranges M (P25, P75) and compared using the Kruskal-Wallis rank sum test. Categorical variables were described as frequencies and percentages, and comparisons were made using Pearson’s chi-squared test or Fisher’s exact test, as appropriate. Restricted cubic spline (RCS) regression models were applied to assess the age-specific positive rates of ALRI and to calculate the odds ratios (OR) for positivity across different pathogens, with 4 knots placed at the 5%, 35%, 65%, and 95% percentiles. RCS analyses were performed using the “rms” package in R software version 4.0.5 (R Foundation for Statistical Computing, Auckland, New Zealand) with 3 degrees of freedom to balance flexibility and overfitting avoidance. A two-sided *P*-value of <0.05 was considered statistically significant.

## 3 Result

### 3.1 General clinical characteristics of patients

A total of 191,967 pediatric ARTI cases were included in this study. Details are shown in **Table 1**. The number of ARTI cases increased year by year from 2020 to 2023, peaking at 50,364 cases in 2023, followed by a slight decline to 45,562 cases in 2024. Male patients accounted for 53.06% of the cohort, consistent with the general population structure in China, with no statistically significant differences across the study years (*P* = 0.131). Among the included ARTI cases, 39.07% were diagnosed with ALRI, and the annual ALRI infection rates differed significantly (*P* < 0.001), with the highest rate observed in 2021 (43.19%), followed by 2024 (40.95%), 2022 (40.74%), and 2020 (38.10%). Additionally, 4.32% of ARTI patients required hospitalization due to disease severity. Seasonality also significantly influenced ARTI incidence (*P* < 0.001), with the highest number of cases reported in autumn (28.25%) and the lowest in summer (22.82%). The median age of the patients was 7 years, and significant differences in age distribution were observed across different years (*P* < 0.001), with the proportion of patients aged 6–14 years declining progressively from 77.91% to 53.53%.

**Table 1:** General information of ARTI from 2020 to 2024.

Characteristics	Overall	2020	2021	2022	2023	2024	*P*-value
	n=191967 (%)	n=21127 (%)	n=34733 (%)	n=40181 (%)	n=50364 (%)	n=45562 (%)	
Sex							0.131
Male	101856(53.06)	11315(53.56)	18446(53.11)	21451(53.39)	26647(52.91)	23997(52.67)	
Female	90111(46.94)	9812(46.44)	16287(46.89)	18730(46.61)	23717(47.09)	21565(47.33)	
Disease							<0.001
AURI	116966(60.93)	13077(61.90)	19732(56.81)	23812(59.26)	33440(66.40)	26905(59.05)	
ALRI	75001(39.07)	8050(38.10)	15001(43.19)	16369(40.74)	16924(33.60)	18657(40.95)	
Type of patients							<0.001
Outpatient	183665(95.68)	20132(95.29)	33521(96.51)	38886(96.78)	48225(95.75)	42901(94.16)	
Inpatient	8302(4.32)	995(4.71)	1212(3.49)	1295(3.22)	2139(4.25)	2661 (5.84)	
Season							<0.001
Spring	47168(24.57)	2313(10.95)	7479(21.53)	11141(27.73)	14512(28.81)	11723(25.73)	
Summer	43805(22.82)	3488(16.51)	9245(26.62)	10539(26.23)	10658(21.16)	9875(21.67)	
Autumn	54223(28.25)	8448(39.99)	10681(30.75)	9551(23.77)	16431(32.62)	9112(20.00)	
Winter	46771(24.36)	6878(32.56)	7328(21.10)	8950(22.27)	8763(17.40)	14852(32.60)	
Age group							<0.001
<3 years	13733(7.15)	0(0.00)	13(0.04)	1605(3.99)	4962 (9.85)	7153(15.70)	
3–5 years	56281(29.32)	4666(22.09)	9216(26.53)	12970(32.28)	15411(30.60)	14018(30.77)	
6–14 years	121953(63.53)	16461(77.91)	25504(73.43)	25606(63.73)	29991(59.55)	24391(53.53)	
Age(years)*	7 [5-9]	7[6–9]	7[5–9]	6[5–9]	6 [4–9]	6 [3–8]	<0.001

“*” The Kruskal-Wallis rank sum test was used, and the data are presented as Median [lQR]. For the rest, Pearson’s Chi-squared test was applied, and the data are shown as n (%).

### 3.2 Monthly and age-specific patterns of ARTI

**Figure 1:**
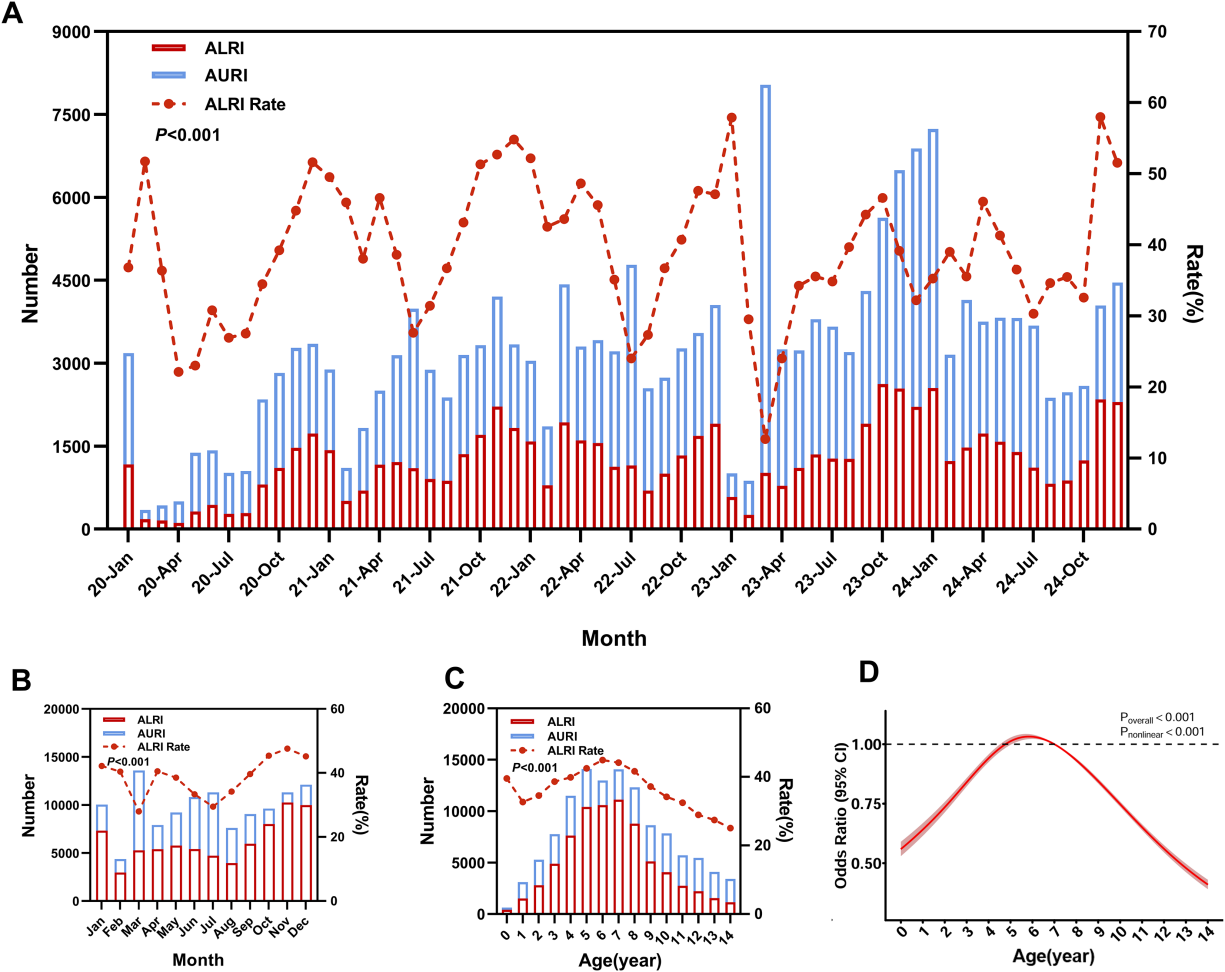
Distribution of ALRI and AURI among children. **(A)** Monthly numbers of ALRI and AURI cases from January 2020 to December 2024 (n=191,967). **(B)** Monthly variation in ALRI and AURI cases across different months (n=191,967). **(C)** Distribution of ALRI and AURI cases across different ages (n=191,967). **(D)** Nonlinear analysis of the risk of ALRI in children aged 0–14 years (n=191,967). The colored shaded areas represent the 95% confidence intervals for the odds ratios (OR). The dotted line represents the reference standard with an OR value of 1.

From 2020 to 2023, the incidence of ARTI in children showed periodic fluctuations. The lowest values in each year occurred in February. However, the epidemic pattern changed in 2024, and the monthly lowest value was postponed to August. A substantial surge in ARTI cases was observed in 2023, with an abnormal peak of 8,035 cases in March, representing a 151.17% increase compared to the overall monthly average (3,199 cases) for 2020–2024 (**Figure 1A**). The number of ALRI cases increased annually, with significant monthly variations (*P* < 0.001). November consistently recorded the highest number and proportion of ALRI cases. In contrast, February had the fewest cases, while March exhibited the lowest infection rate (**Figure 1B**). Age-specific analysis revealed that ALRI cases increased with age from 0 to 6 years, but decreased with age from 7 to 14 years. Notably, the prevalence of AURI also varied significantly across different age groups (*P* < 0.001), as illustrated in **Figure 1C**. To further elucidate the relationship between age and ALRI susceptibility, an RCS regression model was employed. As shown in **Figure 1D**, the age-specific risk curve displayed an unimodal distribution, with the highest susceptibility observed between 5 and 7 years of age, peaking at 6 years. The curve indicated that ALRI incidence rose steadily with age during early childhood (0–6 years) but declined progressively after 6 years of age.

### 3.3 Pathogen detection rates in ARTI

**Figure 2:**
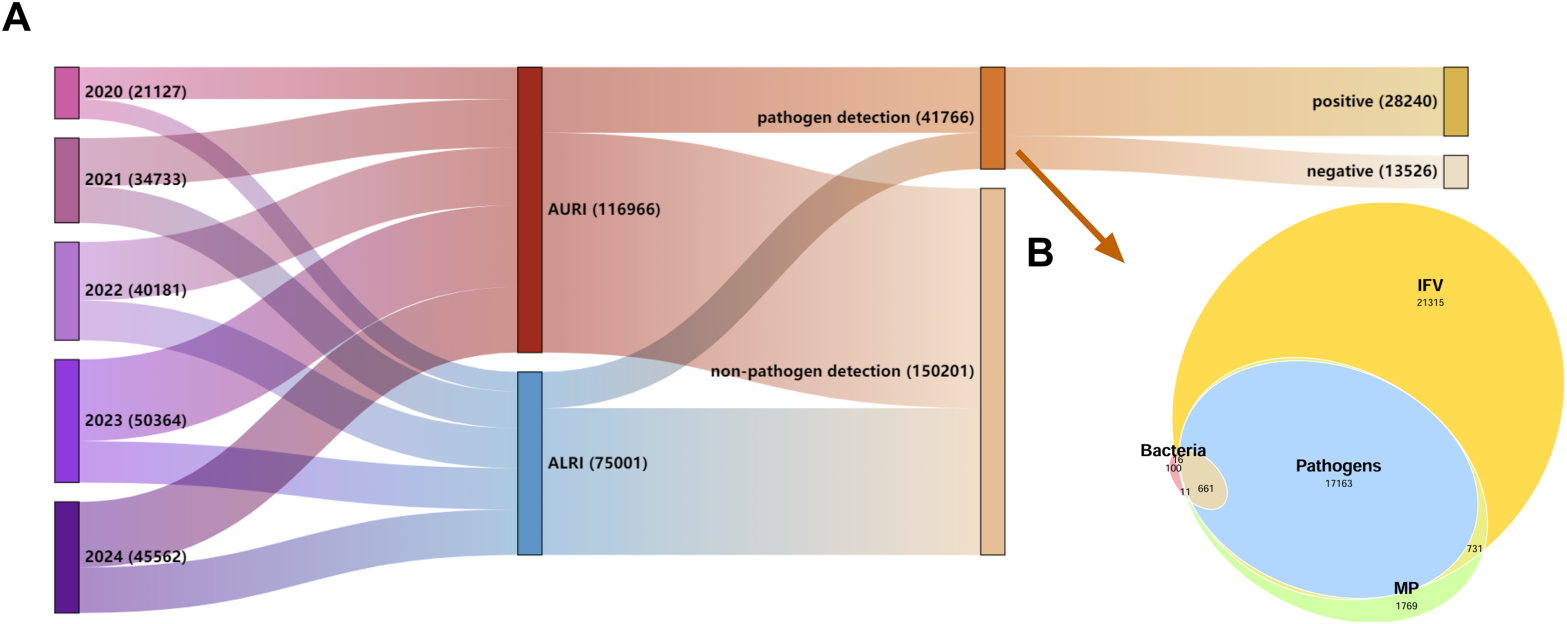
Pathogen detection in acute respiratory tract infections (ARTI). **(A)** Sankey diagram illustrating the number of pathogen tests performed and the corresponding positive detection rates across different disease categories (n=191,967). **(B)** Proportional Venn diagram showing the specific numbers of tests conducted for influenza A/B viruses, *Mycoplasma pneumoniae* (MP), the 13 respiratory pathogens, and bacterial cultures (n=41,766).

Among the 191,967 pediatric ARTI patients, 41,766 (21.76%) underwent pathogen testing. The largest number of tests was conducted for influenza A/B viruses (39,886 cases), followed by *Mycoplasma pneumoniae* (20,335 cases), the 13 respiratory pathogens (17,824 cases), and bacterial culture (788 cases). The overall pathogen detection rate was 67.61% (28,240/41,766), as shown in **Figure 2**. **Table 2** summarizes the detection rates of common pathogens in ARTI cases. Significant differences were observed in the detection rates of IFV-A, IFV-B, HPIV, HMPV, HAdV, HCoV, HRV, HBoV, RSV, CH, MP, and SA between AURI and ALRI cases (*P* < 0.05). Specifically, IFV-A, IFV-B, HPIV, HAdV, and HCoV were more frequently detected in AURI, while HMPV, HRV, HBoV, RSV, CH, MP, and SA were more prevalent in ALRI. Overall, IFV-A had the highest detection rate (31.87%), followed by HRV (24.42%). Among bacteria, SA (22.46%) and HI (8.89%) were the most commonly detected species.

**Table 2:** Detection of different Pathogens in AURI/ALRI.

Pathogens	Overall		AURI		ALRI		*P*-value
	Test	Positive	Test	Positive	Test	Positive	
	n	N (%)	n	N (%)	n	N (%)	
IFV-A	39886	12712(31.87)	26477	10842(40.95)	13409	1870(13.94)	<0.001
IFV-B	39886	3678(9.22)	26477	3010(11.37)	13409	668(4.98)	<0.001
HPIV	17824	1268(7.11)	6830	538(7.88)	10994	730(6.64)	0.002
HMPV	17824	1261(7.07)	6830	312(4.57)	10994	949(8.63)	<0.001
HAdV	17824	2108(11.83)	6830	1257(18.40)	10994	851(7.74)	<0.001
HCoV	17824	523(2.93)	6830	264(3.87)	10994	259(2.36)	<0.001
HRV	17824	4352(24.42)	6830	1492(21.84)	10994	2860(26.01)	<0.001
HBoV	17824	294(1.65)	6830	79(1.16)	10994	215(1.96)	<0.001
RSV	17824	1324(7.43)	6830	217(3.18)	10994	1107(10.07)	<0.001
CH	17824	285(1.60)	6830	72(1.05)	10994	213(1.94)	<0.001
MP	20335	3773(18.55)	7734	619(8.00)	12601	3154(25.03)	<0.001
SP	788	19(2.41)	107	3(2.80)	681	16(2.35)	0.734*
HP	788	17(2.16)	107	1(0.94)	681	16(2.35)	0.493*
HI	788	70(8.89)	107	7(6.54)	681	526(9.25)	0.360
SA	788	177(22.46)	107	10(9.35)	681	167(24.52)	<0.001
MC	788	34(4.31)	107	3(2.80)	681	31(4.55)	0.608*
AB	788	10(1.27)	107	2(1.87)	681	8(1.17)	0.633*
KP	788	30(3.81)	107	4(3.74)	681	26(3.82)	1.000*
EC	788	32(4.01)	107	7(6.54)	681	25(3.67)	0.183*

“*” Fisher’s exact probability method used, and the rest was used Pearson’s Chi-squared test. Data were expressed as n (%).

### 3.4 Monthly-specific patterns of pathogens

**Figure 3:**
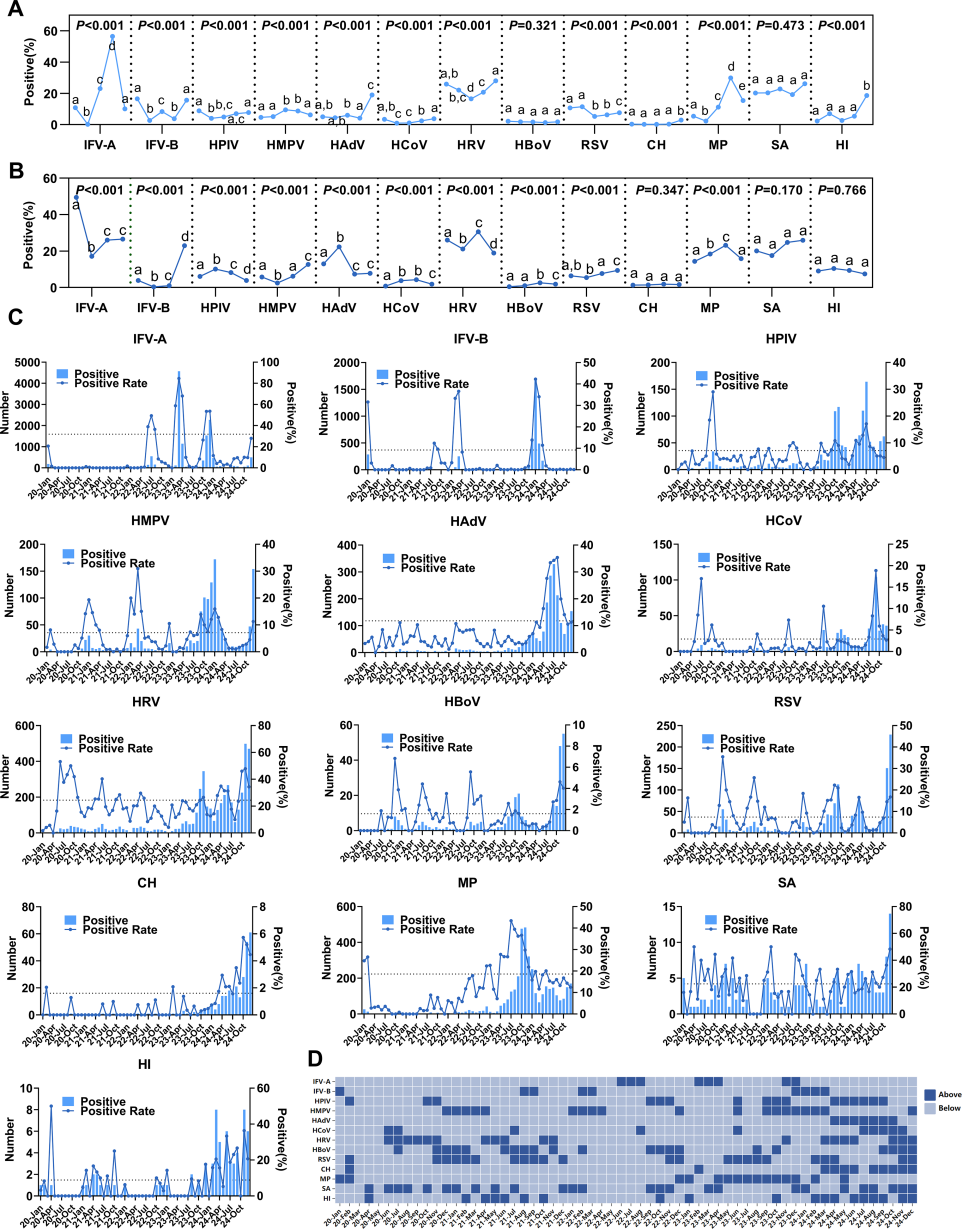
Distribution of common respiratory pathogens. **(A)** Detection rates of each pathogen from 2020 to 2024; the five dots within the dashed boxes represent the detection rates for each year from 2020 to 2024. Different letters indicate that *P* < 0.05 among each subgroup (IFV-A/B, n = 39,886; HPIV, HMPV, HAdV, HCoV, HRV, HBoV, RSV, CH, n = 17,824; MP, n = 20,335; SA/HI, n = 788). **(B)** Seasonal detection rates of each pathogen; the four dots within the dashed boxes represent the detection rates for spring, summer, autumn, and winter, respectively. Different letters indicate that *P* < 0.05 among each subgroup (The data is the same as that in Figure A). **(C)** Monthly detection trends of various pathogens from January 2020 to December 2024, with the black dashed line indicating the overall monthly average detection rate (The data is the same as that in Figure A). **(D)** Summary of pathogen detection trends from January 2020 to December 2024, where dark blue indicates months with detection rates above the overall monthly average and light blue indicates months below the overall average (The data is the same as that in Figure A).

The detection rates of IFV-A, IFV-B, HPIV, HMPV, HAdV, HCoV, HRV, RSV, CH, MP, and HI varied significantly across different years (*P* < 0.05, **Figure 3A**), while HBoV and SA were not significant. Due to low detection numbers, other bacterial species were not included in the analysis. Seasonality was also found to significantly influence pathogen detection. IFV-A, IFV-B, HPIV, HMPV, HAdV, HCoV, HRV, HBoV, RSV, and MP exhibited clear seasonal fluctuations (*P* < 0.05, **Figure 3B**). To further delineate the monthly detection patterns of each pathogen, **Figures 3C, D** were generated. The results showed that, most respiratory pathogens demonstrated significantly higher detection levels in 2023–2024 compared to 2020–2022. Specifically, IFV-A exhibited three epidemic peaks (July 2022, March 2023, and December 2023). IFV-B experienced a minor outbreak in March 2022, followed by a major epidemic in July 2024. HPIV showed bimodal peaks in October 2023 and July 2024, while HMPV peaked in January 2024 and demonstrated another upward trend in December 2024. HAdV and MP experienced concentrated outbreaks in July 2024 and October 2023, respectively. RSV exhibited multiple smaller peaks before a continuous rise at the end of 2024, indicating a potential outbreak risk. Additionally, HBoV and CH showed increased detection toward the end of 2024. It is noteworthy that HRV maintained sustained circulation from 2020 to 2024, with a marked increase in detection after October 2023. Further details for other pathogens are provided in **Figures 3C, D**.

### 3.5 Age-specific patterns of pathogens

**Figure 4:**
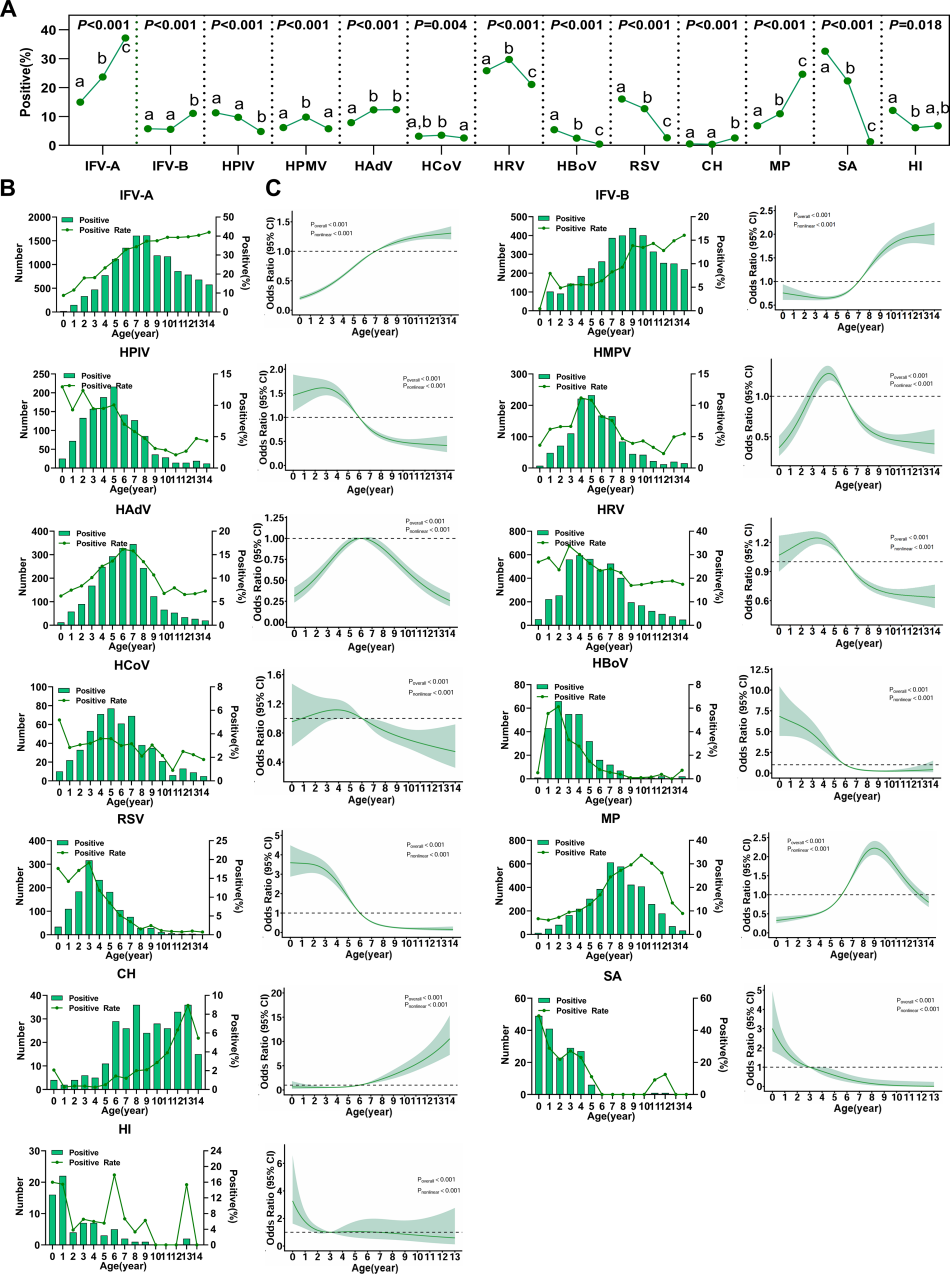
Age-specific detection of common respiratory pathogens. **(A)** Detection rates of each pathogen across different age groups; the three dots within the dashed boxes represent the detection rates for <3 years, 3–5 years, and 6–14 years, respectively Different letters indicate that *P* < 0.05 among each subgroup (IFV-A/B, n = 39,886; HPIV, HMPV, HAdV, HCoV, HRV, HBoV, RSV, CH, n = 17,824; MP, n = 20,335; SA/HI, n = 788). **(B)** Pathogen detection distribution across ages 0–14 years (The data is the same as that in Figure A). **(C)** Nonlinear analysis of infection risk across ages 0–14 years (The data is the same as that in Figure A); the colored shaded areas represent the 95% confidence intervals for the odds ratios (OR). The dotted line represents the reference standard with an OR value of 1.

Except for SA and HI, other bacterial species were excluded from the analysis due to their low detection numbers. The results showed significant differences in the detection rates of all pathogens across different age groups (*P* < 0.05, **Figure 4A**). To further clarify the distribution of pathogens among different age groups, **Figures 4B, C** were generated, revealing nonlinear relationships between pathogen infection rates and age. Specifically, susceptibility to IFV-A, IFV-B, and CH increased with age, while susceptibility to HPIV, RSV, and HBoV decreased as age increased. In contrast, susceptibility to HMPV, HAdV, HRV, and MP first increased and then decreased with age. Most pathogens displayed heightened susceptibility in children under 6 years old, including HPIV, HCoV, HRV, HBoV, RSV, SA, and HI. Conversely, IFV-A, IFV-B, and CH were more prevalent among children aged 6–14 years, with MP showing maximal detection aged 6–13 years. No consistent age-specific pattern was identified for HAdV.

### 3.6 Co-infections

**Figure 5:**
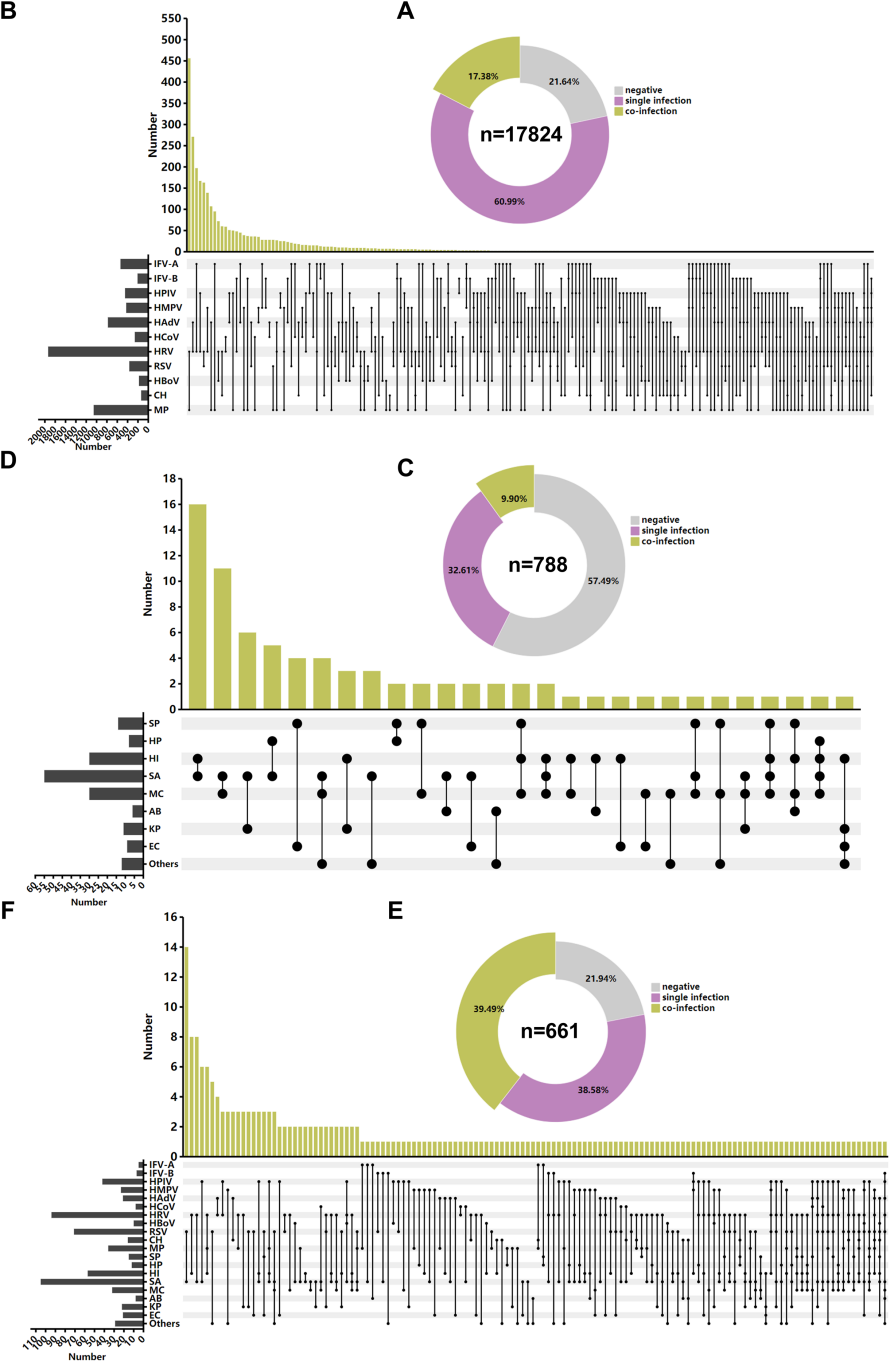
Co-infections among respiratory pathogens. **(A)** Co-infection rates among children tested with the 13 respiratory pathogens group (n=17,824). **(B)** Upset diagram showing the distribution of pathogens involved in co-infections among the 13 respiratory pathogens group (n=17,824). **(C)** Co-infection rates among children who underwent bacterial culture testing (n=788). **(D)** Upset diagram showing the distribution of pathogens involved in co-infections within the bacterial culture testing group (n=788). **(E)** Co-infection rates among children who underwent combined testing with the 13 respiratory pathogens and bacterial culture (n=661). **(F)** Upset diagram showing the distribution of pathogens involved in co-infections within the combined testing group (n=661). In panels **(B)**, **(D)**, and **(F)**, the black bar graphs represent the number of detections for each pathogen involved in co-infections, the black lollipop plots represent different co-infection combinations, and the yellow bar graphs represent the number of cases for each specific co-infection pattern.

Co-infection analysis was conducted by dividing the cases into three groups: the 13 respiratory pathogens group (17,824 cases), the bacterial culture group (788 cases), and the combined testing group involving both 13 respiratory pathogens and bacterial culture (661 cases). In the 13 respiratory pathogens group, 17.38% (3,097 cases) exhibited co-infection with multiple pathogens. The most common co-infection patterns were HRV-MP (456 cases, 14.72%), HRV-IFV-A (271 cases, 8.75%), and HAdV-HRV (197 cases, 6.36%). In the bacterial culture group, 9.90% (78 cases) showed co-infections, with the most frequent combinations being HI-SA (16 cases, 20.51%), SA-MC (11 cases, 13.92%), and SA-KP (6 cases, 7.59%). Among patients who underwent both pathogen panel and bacterial culture testing, the co-infection rate was as high as 39.49% (261 cases). The most common co-infection patterns in this group were SA-RSV (14 cases, 5.36%), HI-HRV (8 cases, 3.07%), and SA-RV (8 cases, 3.07%). Details are illustrated in **Figure 5**.

### 3.7 Pathogen correlations

**Figure 6:**
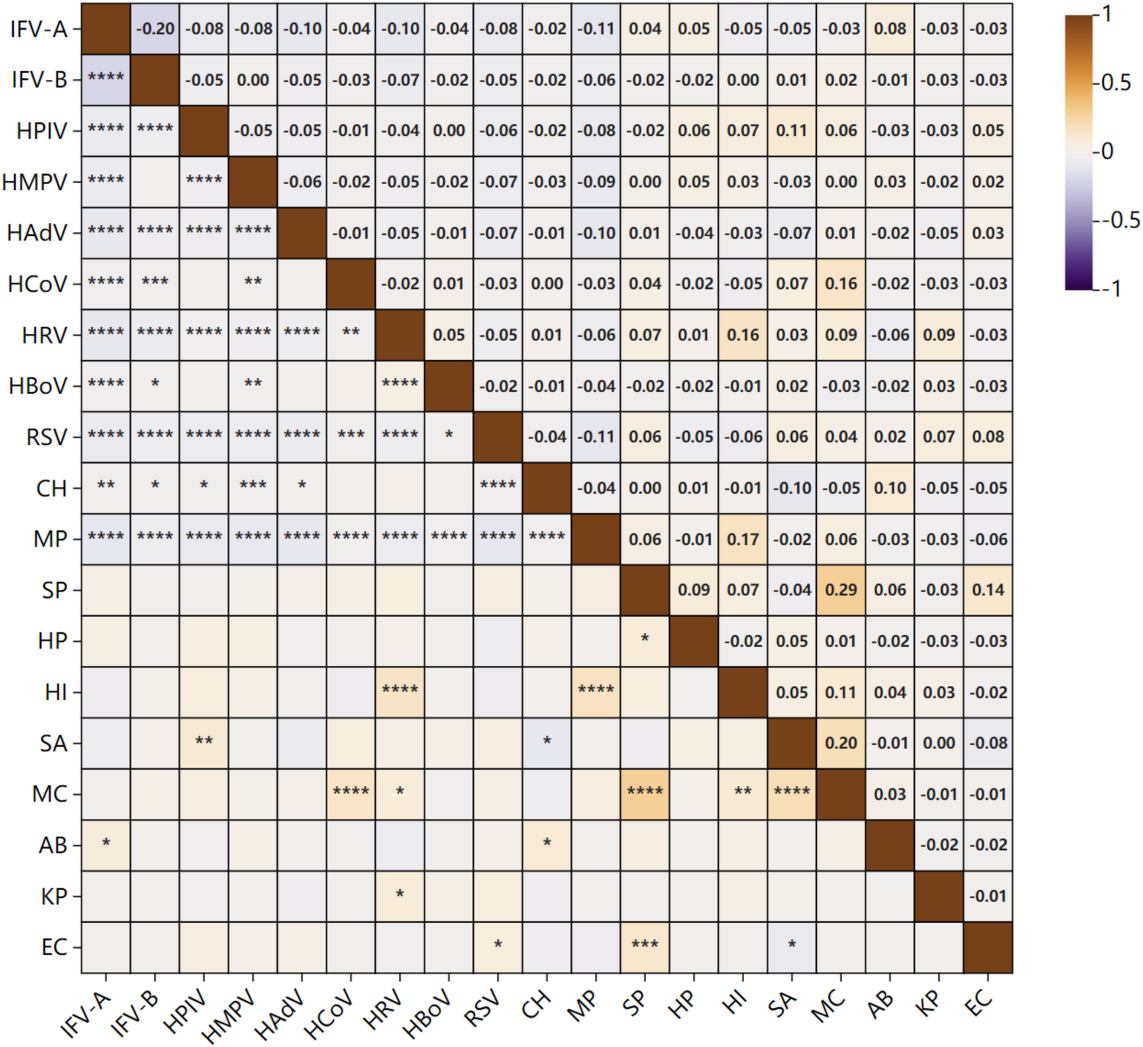
Correlations among respiratory pathogens. The lower triangular matrix indicates the *P*-values for correlations between pathogens on the x-axis and y-axis (n=41,766). “****” represents *P* < 0.0001, “***” represents *P* < 0.001, “**” represents *P* < 0.001, “*” represents *P* < 0.05, and blank cells indicate no statistically significant difference between the two groups. The upper triangular matrix shows the correlation coefficients between pathogens: values closer to “1” indicate stronger synergistic interactions, and values closer to “-1” indicate stronger antagonistic interactions, values near 0 indicate no significant correlation.

To explore the interactions between respiratory pathogens, cross-analysis was conducted on the 41,766 specimens subjected to pathogen testing (**Figure 6**). The results showed that virus-virus interactions were predominantly negative, with only a weak positive correlation observed between HRV and HBoV. Antagonistic relationships were evident among multiple viruses, including IFV-A, IFV-B, HPIV, HRV, and RSV. In contrast, bacterial interactions were relatively sparse but mostly positive, such as those between SP-EC, SP-MC, and MC-HI, as well as MC-SA. However, a competitive relationship was identified between EC and SA. Positive correlations were also more common between viruses and bacteria, such as HPIV-SA, HCoV-MC, HRV-HI, and HRV-MC. Moreover, MP and CH showed notable associations with multiple pathogens: MP demonstrated a positive correlation with HI, but negative correlations with IFV-A, IFV-B, HPIV, HRV, and HAdV. CH exhibited a positive correlation with AB, negative correlations with SA, and antagonistic relationships with multiple viruses including IFV-A, IFV-B, HPIV, and RSV. Despite the presence of various associations among pathogens, correlation coefficient analysis indicated that the overall strength of these interactions was generally weak.

## 4 Discussion

From 2020 to 2024, there were significant changes in the incidence of pediatric acute respiratory tract infections (ARTI) and the distribution of common pathogens in Ningbo. These trends were influenced by multiple factors, including alterations in host immune status, environmental exposures, pathogen evolution, and adjustments to global public health policies.

This study found that the number of ARTI cases continuously increased from 2020 to 2023, reaching a peak in 2023, and subsequently stabilizing in 2024. On January 20, 2020, Ningbo implemented extensive non-pharmaceutical interventions (NPIs), including universal masking, social distancing, and restrictions on gatherings (Hsiang et al., 2020; Ding and Zhang, 2022). These measures effectively controlled and even temporarily suppressed the COVID-19 outbreak, while also leading to a dramatic decline in ARTI cases (from 3,182 cases in January 2020 to 348 cases in February 2020). On December 9, 2022, China announced the optimization and adjustment of COVID-19 prevention and control measures, and on January 8, 2023, China downgraded the management of COVID-19 from a Category A to a Category B infectious disease (The Lancet Regional Health-Western, 2023). Following this shift, the incidence of pediatric ARTI surged sharply, with the number of infections in 2023 reaching 238% of the 2020 baseline. “Immunity debt” might be one of the main factors contributing to this phenomenon. During the three-year implementation of NPIs, the insufficient stimulation of pathogens to children has weakened their humoral immunity, leading to an increase in the susceptible population (Liu et al., 2024). When NPIs were lifted, the relatively vulnerable immune systems of children could not effectively defend against circulating pathogens, resulting in large-scale outbreaks of ARTI. Similar patterns have been observed in multiple countries and region (Chan et al., 2021; Amar et al., 2022; Wang et al., 2022; Singer et al., 2024).

Subsequent retrospective analysis of pathogen detection data revealed that IFV-A, IFV-B, HPIV, HAdV, and HCoV were more frequently detected in AURI cases, while HMPV, HRV, HBoV, RSV, CH, MP, and SA were more commonly associated with ALRI. This distinct distribution pattern is closely linked to the molecular pathogenic mechanisms of each pathogen. For instance, influenza viruses exhibit strong epithelial tropism; their hemagglutinin (HA) proteins specifically recognize sialic acid receptors on the surface of ciliated epithelial cells in the nasopharynx, resulting in classic symptoms such as nasal congestion and sore throat (Bui et al., 2019). Conversely, RSV and HMPV possess fusion (F) proteins that directly act on alveolar epithelial cells, leading to bronchiolitis and pneumonia (Shibata et al., 2020; Papenburg and Alghounaim, 2021). Surveillance data from 18 hospitals also indicated that the detection rates of IFV and HPIV were higher in AURI than in ALRI cases (Huang et al., 2020), consistent with our findings.

This study further demonstrated that detection rates of IFV-A, IFV-B, HPIV, HMPV, HAdV, HCoV, HRV, RSV, CH, MP, and HI markedly increased in 2023–2024 compared to 2020–2021, while HBoV and SA detection rates did not exhibit significant changes. During 2020–2021, influenza virus activity was notably suppressed under stringent NPIs. Following the easing of NPIs, IFV-A displayed a double-peak pattern, with surges observed in March and December 2023. Similarly, IFV-B experienced a minor epidemic in March 2022 before a larger outbreak occurred in July 2024. These epidemic patterns closely mirror surveillance reports from other cities in China, such as Xiamen and Shijiazhuang (Zheng et al., 2024; Li et al., 2025). International data also confirmed that influenza virus activity remained historically low during the COVID-19 pandemic in countries like the United States, Canada, and Australia, but rebounded rapidly after the relaxation of public health measures (Olsen et al., 2020, Olsen et al., 2021; Park et al., 2021). Additionally, MP outbreaks were noted during the spring and summer of 2023, with continued detection into 2024, consistent with trends observed in Guangzhou and Chongqing (Xu et al., 2024; You et al., 2024). A sharp increase in HAdV infections was reported in South Korea by the end of 2023, with a positivity rate of 42.2% (Lee et al., 2024), and in southern China in early 2024, with a positivity rate of 10.75% (Wang et al., 2024). Our study found that HAdV detection began to rise at the end of 2023 but peaked in July 2024, reaching a detection rate of 34.31%. This discrepancy may be attributed to differences in monitoring periods or regional variations. Moreover, HMPV exhibited a significant detection surge in early 2024, peaking in January and resurging in December, while RSV maintained multiple minor peaks from 2020 to 2024 and showed a continuous rise at the end of 2024, signaling a potential risk for future outbreaks. The Chinese Center for Disease Control and Prevention (Al-Tawfiq and Memish, 2025) also reported increased detection rates of HMPV and RSV in December 2024, although still within expected levels, warranting continued vigilance. Of note, HRV remained persistently prevalent during the pandemic period, with a substantial increase in detection after October 2023. This trend may be associated with the biological characteristics of non-enveloped viruses like HRV, which can rapidly rebound and maintain high activity levels once NPIs are lifted. Monitoring data from the United States, Australia, Guangzhou (China), and Brazil support this conclusion (Ding and Zhang, 2022; Regina Malveste Ito et al., 2024).

From the perspective of the seasonal characteristics of pathogen epidemics, except for CH, SA, and HI, most respiratory pathogens show obvious seasonal sensitivity. This phenomenon may be related to the periodic fluctuations of the host’s physiological state caused by climate change and may also be associated with the seasonal differences in population patterns. Among many external driving factors, climatic conditions are considered one of the key factors for the seasonal changes in respiratory infections caused by pathogens (Moriyama et al., 2020). Existing studies have confirmed that a cold and dry environment helps to improve the stability of viruses and promote their spread, especially having a more significant impact on enveloped viruses such as influenza viruses and RSV (He et al., 2023). Recent studies have shown that there may be a non-linear relationship between meteorological variables and the prevalence of respiratory pathogens. In different climatic regions, both “cold and dry” and “warm and humid” environments may trigger the peak prevalence of pathogens (Xu et al., 2021). Further systematic studies across regions and involving multiple pathogens are needed to reveal the complex interaction mechanisms between meteorological factors and the spread of respiratory pathogens.

Multiple respiratory pathogens exhibit significant differences in susceptibility characteristics among different age groups. This difference stems from both the unique transmission mechanisms and pathogenic characteristics of the pathogens and is closely related to the age-related immune development patterns in children. Young children, due to their immature immune systems and the gradual decline of maternally-transmitted antibodies, combined with the high-density exposure in childcare institutions, often become the main targets of most respiratory pathogens. Although school-age children have enhanced resistance to certain pathogens due to the accumulation of immune memory, they become high-risk groups for specific pathogens such as influenza viruses and MP because of their expanded social scope and immune response characteristics (Sun et al., 2024). This study also found that the susceptibility to ALRI exhibited a nonlinear relationship with age, with a high-risk window between 5 and 7 years, and the highest susceptibility observed at 6 years of age. This phenomenon may result from the dynamic imbalance among three factors: the waning of maternal antibody protection, increased pathogen exposure due to social interactions at school, and the rapid yet incomplete maturation of the child’s immune system (Kloc et al., 2020). Further age-specific analysis revealed that different pathogens displayed distinct age-related susceptibility patterns. The susceptibility to IFV-A, IFV-B, and CH increased progressively with age, while susceptibility to HPIV, RSV, and HBoV declined with age. For pathogens such as HMPV, HAdV, HRV, and MP, susceptibility first increased and then decreased as children aged. These patterns are closely associated with the biological characteristics of the respective pathogens. For example, influenza viruses can undergo antigenic drift, enabling continuous immune evasion and leading to repeated infections, while RSV infection can confer more lasting immune protection. In the case of MP, the biphasic pattern may be explained by the maturation of mucociliary function during school-age years, which initially facilitates pathogen adhesion and infection, followed by the gradual strengthening of immune defenses during adolescence (Chow et al., 2023). Similar age-susceptibility patterns for pathogens have been observed in other studies by Li (Li et al., 2021) and Wang (Wang et al., 2022). Based on these findings, future clinical practice should incorporate age-based surveillance mechanisms and targeted immunization strategies to more effectively prevent infections caused by specific pathogens and reduce the burden of ALRI.

Co-infections were found to be highly diverse and frequent, posing considerable challenges to clinical diagnosis and treatment. To elucidate the co-infection patterns in Ningbo, cases were categorized into three groups. Among the 13 respiratory pathogens group, 17.38% of children exhibited co-infections, with the most common combination being HRV-MP. In the bacterial culture group, 9.90% of children were co-infected, most frequently with HI-SA. In the combined testing group (both 13 respiratory pathogens group and bacterial culture), the co-infection rate reached as high as 39.49%, with SA-RSV being the most common combination. The co-infection rate observed in our 13 respiratory pathogens group (17.38%) was comparable to that reported in Xiamen (16.79%) (Li et al., 2025), but notably higher than that in Shandong Province (4.69%) (Zhang et al., 2023). A multi-center study involving 106 cities reported a co-infection rate of 34.3% among children who underwent simultaneous bacterial and viral testing (Li et al., 2021), slightly lower than our findings. Co-infections are clinically heterogeneous and highlight the urgent need for the development of rapid and precise multiplex pathogen detection systems. Such systems could provide crucial technical support for early differential diagnosis and individualized treatment strategies.

Previous studies have reported interactions among respiratory pathogens (Chow et al., 2023). In this study, we found that most virus-virus interactions were antagonistic, particularly among IFV-A, IFV-B, RSV, and HAdV. In contrast, interactions between bacteria and viruses were predominantly synergistic, such as those between HI and HRV, and between RSV and EC. Similar findings were reported by Li (Li et al., 2021). The mechanisms underlying virus-virus antagonism may involve immune activation processes, such as interferon responses that inhibit the replication of other pathogens, as well as competition for cellular resources (Essaidi-Laziosi et al., 2020). Regarding bacterial-viral synergism, Shibata (Shibata et al., 2020) found that RSV infection could impair bacterial clearance, thereby enhancing susceptibility to bacterial pathogens. Kloepfer (Kloepfer and Kennedy, 2023) also demonstrated that *Streptococcus pneumoniae* could facilitate viral replication in the respiratory tract. Although various pathogen interactions were observed, correlation strength analysis revealed that the overall associations were generally weak. This suggests that pathogen interactions are likely modulated by multiple factors, including host immune status and seasonal fluctuations. Currently, the underlying mechanisms governing these interactions remain incompletely understood and need to be verified by subsequent experimental studies (such as *in vitro* co-culture and animal models).

This study has certain limitations. As a single-center retrospective study, although 191,967 cases of acute respiratory infections were included, the cohort only covered children who sought medical treatment and did not include those with mild symptoms or self-limiting conditions who did not seek medical help. Secondly, pathogen detection was based on clinical indications rather than random sampling. This may overestimate the epidemiological importance of pathogens associated with severe illness, such as influenza virus and *Mycoplasma pneumoniae*, while underestimating the spread of common mild pathogens, such as rhinovirus and seasonal coronaviruses. In addition, the distinction between upper and lower respiratory tract infections relied on clinical diagnosis rather than the gold standard of etiology or imaging, which may lead to misclassification bias. Despite efforts to exclude COVID-19 cases, it was still impossible to completely avoid the inclusion of asymptomatic or undiagnosed SARS-CoV-2 infected individuals, their prevalence, and the possible immune interference they may cause. Finally, due to the lack of information regarding vaccination history (such as influenza vaccine) and pre-admission medication use, it is difficult to evaluate the potential impact of immunization on the transmission dynamics of pathogens. Moreover, it is impossible to completely rule out the interference of previous drug treatment on the pathogen detection results.

In summary, through the analysis of clinical big data from the past five years, this study reveals that the epidemiological characteristics of ARTI in children in the Ningbo area are gradually returning from the abnormal quiescent state during the COVID-19 pandemic to the normal mode of co-circulation of multiple pathogens, and show significant age and season specificity. Despite the inherent limitations of retrospective studies, our findings still provide important insights into understanding the changes in the pathogen spectrum of childhood respiratory infections in the post-pandemic era, highlighting the value of continuous multi-pathogen monitoring and age-directed prevention and control strategies.

## 5 Conclusion

Pediatric ARTI exhibit marked age-dependent susceptibility, with distinct age-specific vulnerability patterns across pathogens and complex inter-pathogen interactions. The 2023–2024 ARTI resurgence underscores the critical importance of elucidating these epidemiological dynamics for developing precision prevention strategies, providing essential evidence for optimizing targeted interventions.

## Data Availability

The datasets presented in this study can be found in online repositories. The dataset supporting this study is openly available in FigShare at https://doi.org/10.6084/m9.figshare.29045306.
