# Supplementary material for: Multi-dimensional epidemiology of pediatric acute respiratory tract infection in Ningbo, China (2020–2024): age-specific susceptibility, pathogen dynamics, and epidemiological trends
Source: Front Cell Infect Microbiol. 2026 Feb 2;16:1662777. doi: 10.3389/fcimb.2026.1662777 (PMC12907365; doi:10.3389/fcimb.2026.1662777)
Supplement: Supplementary file 1 [file Table1.docx]

**Supplemental materials**

1. **Different Polymerase Chain Reaction (PCR) protocols of different kits**

**Supplementary Table 1 PCR amplification procedure for influenza A/B viruses**

| **Step** | **Temperature** | **Time** | **Cycles** |
| --- | --- | --- | --- |
| 1 | 50℃ | 5 min | 1 |
| 2 | 95℃ | 1 min | 1 |
| 3 | 95℃ | 10 sec | 41 |
|  | 60℃ | 20 sec |  |

**Supplementary Table2 PCR amplification procedure for *Mycoplasma pneumoniae***

| **Step** | **Temperature** | **Time** | **Cycles** |
| --- | --- | --- | --- |
| 1 | 50℃ | 2 min | 1 |
| 2 | 95℃ | 2 min | 1 |
| 3 | 91℃ | 15 sec | 40 |
|  | 64℃ | 1 min |  |

**Supplementary Table 3 PCR amplification procedure for 13 respiratory pathogens**

| **Step** | **Temperature** | **Time** | **Cycles** |
| --- | --- | --- | --- |
| 1 | 25℃ | 5 min | 1 |
| 2 | 50℃ | 15 min | 1 |
| 3 | 95℃ | 2 min | 1 |
| 4 | 94℃ | 30 sec | 65→60°C, annealing with a 1°C drop per cycle, 6 cycles |
|  | 65→60℃ | 30 sec |  |
|  | 72℃ | 60 sec |  |
| 5 | 94℃ | 30 sec | 29 |
|  | 60℃ | 30 sec |  |
|  | 72℃ | 60sec |  |
| 6 | 72℃ | 10min | 1 |
| 7 | 4℃ | - | 1 |

1. **The product performance of different kits**
   1. **influenza A/B viruses（Sansure Biotech, Changsha, China）**

（1）Accuracy

When testing the positive reference products of the enterprise, all the results were positive.

（2）Specificity

This kit has no cross-reaction with positive samples of coronaviruses (NL63, HKU1, 229E, OC43), SARS-coronavirus, MERS-coronavirus, respiratory syncytial virus types A and B, rhinovirus types A, B, and C, adenovirus types 1, 2, 3, 4, 5, 7, 55, parainfluenza virus types 1, 2, 3, enterovirus types A and B, enterovirus C (EV.C95), enterovirus D (EV-D70), metapneumovirus, human interstitial pneumovirus, *Cryptococcus neoformans*, *Streptococcus pyogenes,* *Acinetobacter baumannii*, *Pneumocystis jirovecii*, *Klebsiella pneumoniae*, *Streptococcus pneumoniae*, *Haemophilus influenzae*, *Pseudomonas aeruginosa*, *Legionella pneumophila*, *Bordetella pertussi*s, *Staphylococcus aureus*, *Mycoplasma pneumoniae*, *Chlamydia pneumoniae*, Epstein-Barr virus, human cytomegalovirus, *Aspergillus fumigatus*, *Candida albicans*, *Candida glabrata*, *Mycobacterium tuberculosis*, *non-tuberculous mycobacteria*, Shiru virus, rotavirus, varicella-zoster virus, measles virus, mumps virus, human genomic DNA, Bocavirus, *Neisseria meningitidis*, *Staphylococcus epidermidis,* *Pneumocystis*, *Corynebacterium diphtheriae*, *Streptococcus salivarius*, *Lactobacillus bulgaricus*, *Moraxella catarrhalis*, etc.

1. Limit of detection

The limit of detection for both influenza A virus and influenza B virus is 200 copies/mL.

1. Precision

The coefficient of variation (CV, %) of the Ct values in intra-batch/inter-batch and intra - day/inter-day tests is ≤5%.

- 1. ***Mycoplasma pneumoniae*（Mole Bioscience, Jiangsu, China)**

（1）Accuracy

When testing the positive reference products of the enterprise, all the results were positive, and when testing the negative reference products, all the results were negative.

（2）Specificity

There is no cross-reaction with other pathogens with the same infection site or similar infection symptoms (such as varicella-zoster virus, influenza A virus, influenza B virus, adenovirus, herpes simplex virus, rhinovirus, hepatitis B virus, hepatitis C virus, *Chlamydia pneumoniae*, *streptococcus*, *Staphylococcus aureus*, and *pneumococcus*).

1. Limit of detection

For the limit of detection reference products MM2063 and MM2064L1-L2, *Mycoplasma pneumoniae* and drug-resistant positive should be completely detected. L3 can detect *Mycoplasma pneumoniae* and drug-resistant positive, and L4 can detect *Mycoplasma pneumoniae* and drug-resistant negative. Among them, the copy number of the target gene corresponding to L1 is 50000 copies/mL, the concentration of the target gene corresponding to L2 is 5000 copies/mL, and the concentration of the target gene corresponding to L3 is 500 copies/mL.

1. Precision

The coefficient of variation (CV, %) of the Ct values is ≤5%.

- 1. **13 respiratory pathogens(****Health Genetech, Ningbo, China）**

（1）Accuracy

**Nin**

When testing the positive reference products of the enterprise, all the results were positive, and when testing the negative reference products, all the results were negative.

（2）Specificity

There is no cross-reaction with positive samples of the following pathogens at the given concentrations: cytomegalovirus at 7.5*10⁶ Copies/mL, *Streptococcus pyogenes* at 1.5*10⁸ CFU/mL, *Klebsiella pneumoniae* at 7.3*10⁷ CFU/mL, *Ureaplasma urealyticum* at 2.7*10⁵ Copies/mL, *Staphylococcus epidermidis* at 6.2*10⁸ CFU/mL, *Escherichia coli* at 5.8*10¹¹ CFU/mL, Epstein-Barr virus at 1.4*10⁹ Copies/mL, *Enterococcus faecalis* at 2.6*10⁹ CFU/mL, *Staphylococcus aureus* at 5.0*10⁸ CFU/mL, *Pseudomonas aeruginosa* at 3.0*10⁹ CFU/mL, national reference products of rubella virus, varicella-zoster virus, measles virus, *Pseudomonas aeruginosa*, mumps virus, and *Escherichia coli*.

1. Limit of detection

Influenza A virus: The limit of detection is 0.098 TCID₅₀/mL; H1N1 (2009): 0.098 TCID₅₀/mL; H3N2: 0.1 TCID₅₀/mL; influenza B virus (Victoria and Yamagata): 2.0 TCID₅₀/mL; respiratory syncytial virus (groups A and B): 0.4 TCID₅₀/mL; parainfluenza virus (types 1, 2, 3, 4): 0.4 TCID₅₀/mL; coronaviruses (OC43, HKU1, NL63, 229E): 0.3 TCID₅₀/mL; rhinovirus: 0.15 TCID₅₀/mL; metapneumovirus: 0.35 TCID₅₀/mL; Bocavirus: 5,000 Copies/mL; adenovirus: 2,000 Copies/mL or 1 TCID₅₀/mL; *Chlamydia*: 5,000 Copies/mL; *Mycoplasma pneumoniae*: 3,000 Copies/mL.

1. Repeatability

When the repeatability reference product is tested 10 times repeatedly, the results are consistent.

- 1. **Mass spectrometry system(Zhongyuan Huiji Biotechnology, Chongqing, China)**

（1）Accuracy

Positive test: Use this product to process the standard strains *Enterococcus faecium* (ATCC700221), *Escherichia coli* (ATCC25922), and *Candida albicans* (ATCC10231) to obtain positive samples. When identified by the microbial mass spectrometry detection system, the results should correctly identify the species and genus. Negative test: Take 1 μL of the matrix solution and drop it on the target plate. After drying, use the microbial mass spectrometry calibration and detection system for identification. The result should be negative.

（2）Limitations

①For the bacterial species that cannot be effectively distinguished due to the limitations of the mass spectrometry-based microbial identification method itself, using this kit cannot effectively improve the identification results.

②This method only identifies the microorganisms that have been entered into the database.

If there are two or more types of microorganisms in the sample, using this product cannot complete accurate identification.

③When the sample collection amount is too large, the cell-wall breaking effect of the microorganisms may be reduced, thus affecting the identification results.

④When the sample collection amount is too small (below the sensitivity of the microbial mass spectrometer), the identification results will be affected.
